# Hydrops Gallbladder Caused by Cystic Duct Fibrosis Leading to Mirizzi Syndrome: A Case Report

**DOI:** 10.7759/cureus.68328

**Published:** 2024-08-31

**Authors:** Bipin Adhikari, Luis M Nieto, Biplab Adhikari, Alina Dhital, Cinna Attar

**Affiliations:** 1 Internal Medicine, Wellstar Spalding Medical Center, Griffin, USA; 2 Gastroenterology and Hepatology, Emory University, Atlanta, USA; 3 Infectious Diseases, School of Medicine, University of Louisville, Louisville, USA; 4 Internal Medicine, Essen Health Care, New York, USA; 5 Hospital Medicine, Wellstar Spalding Medical Center, Griffin, USA

**Keywords:** magnetic resonance cholangiopancreatography, laparoscopic cholecystectomy, obstructive jaundice, cystic duct fibrosis, hydrops gallbladder, mirizzi syndrome

## Abstract

Mirizzi syndrome (MS) is a rare complication of cholelithiasis, resulting from the extrinsic compression of the common hepatic duct or common bile duct by impacted gallstones in the cystic duct or Hartmann's pouch. MS is most commonly observed in the elderly with a long-standing history of gallstones.

We present the case of MS type I diagnosed following magnetic resonance cholangiopancreatography (MRCP). Surgical management was performed with laparoscopic cholecystectomy.

MS should be considered as a differential diagnosis in elderly patients presenting with asymptomatic obstructive jaundice. Imaging studies such as MRCP and endoscopic retrograde cholangiopancreatography (ERCP) are essential for diagnosing. We present this case to highlight the importance of recognizing hydrops gallbladder caused by cystic duct fibrosis leading to MS.

## Introduction

Mirizzi syndrome (MS), first described by Kehr in 1905 and Ruge in 1908, was later named by Pablo Luis Mirizzi in 1948 [1]. This complication occurs commonly when gallstones become lodged in the cystic duct or the neck of the gallbladder (Hartmann’s pouch). This impaction leads to gallbladder dilation, which externally compresses the common bile duct or common hepatic duct, leading to obstructive jaundice. Eventually, the stone can erode and create a cholecystocholedochal fistula [2]. The infrequent and complex presentation of MS makes it particularly challenging to diagnose. It often mimics other hepatobiliary disorders. Delayed treatment can result in complications such as biliary fistula formation, cholangitis, and biliary cirrhosis [3].

When assessing an elderly patient with symptoms of obstructive jaundice, considering MS as a potential diagnosis is crucial along with other probable diagnoses that include gallbladder cancer, cholangiocarcinoma, pancreatic cancer, sclerosing cholangitis, and metastatic disease commonly involving liver and pancreas [4]. We present a rare case of MS with hydrops gallbladder caused due to fibrosis of the cystic duct that led to obstructive features.

## Case presentation

A 79-year-old man came to the emergency department after falling and hitting his right hip while using an electric chainsaw on a golf cart. He reported severe pain in his right hip (rated 7/10) and was unable to walk post-fall. His medical history included chronic obstructive pulmonary disease (COPD), inflammatory bowel disease (ulcerative colitis), right colostomy due to colitis, and chronic kidney disease (CKD) stage 4. The patient added that there had been a change in urine color to dark and pale stool for a week.

**Figure 1:**
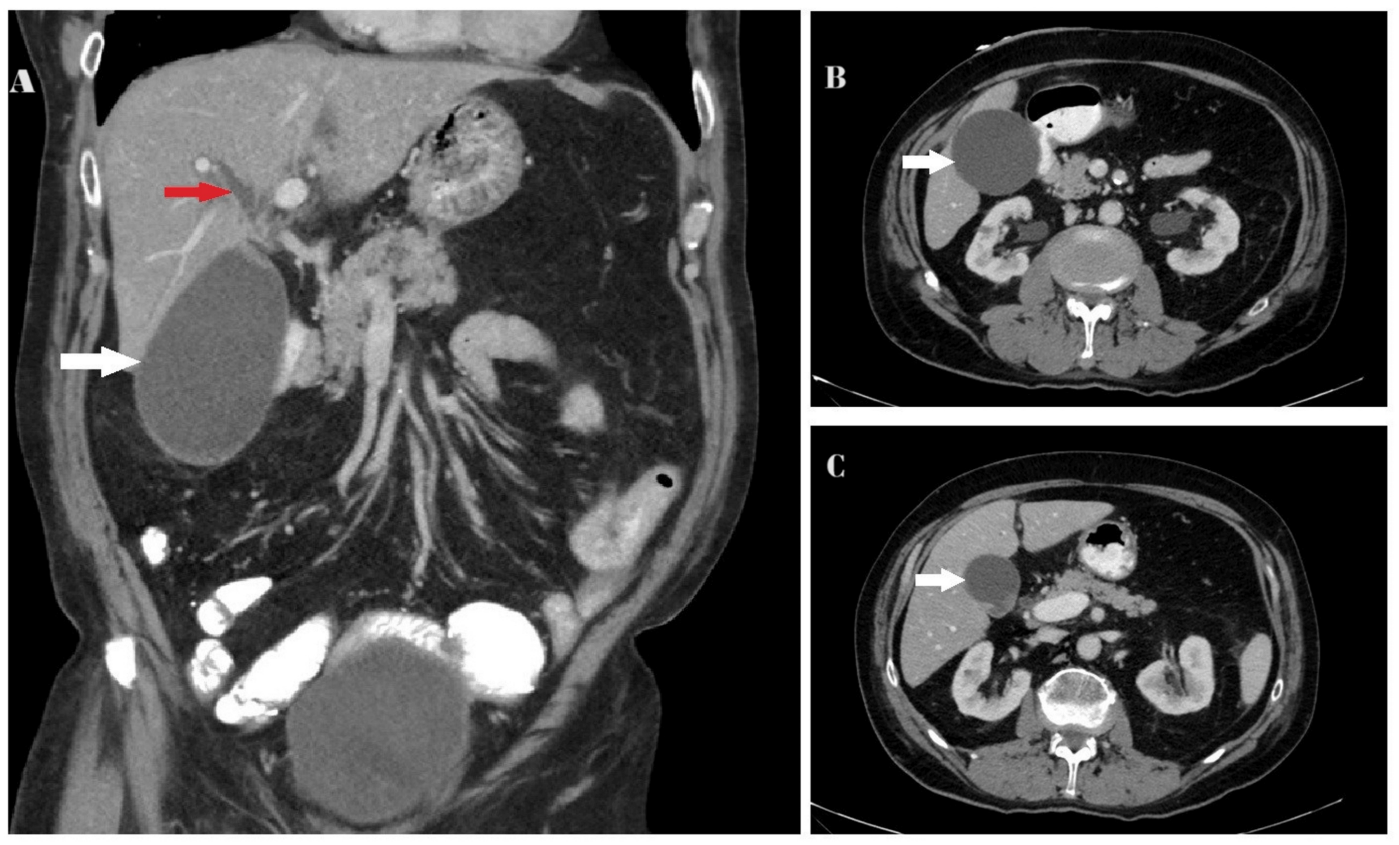
CT scan images of the abdomen and pelvis (A) Coronal view showing marked gallbladder distention (white arrow) and early intrahepatic biliary dilatation (red arrow). (B) Axial view, white arrows indicating gallbladder distension at the level of T12. (C) Axial view, white arrows indicating gallbladder distension at the level of L2 CT, computed tomography; L2, lumbar vertebral 2; T12, thoracic vertebral 12

During the physical examination, there was erythema, swelling, tenderness, and a limited range of motion in his right hip. Additionally, yellowing of the sclera, mild jaundiced skin with occasional pruritus, and mucous membranes were noticed. All other physical assessments were unremarkable. Pain management interventions were initiated promptly. X-ray and computed tomography (CT) scan revealed a closed femoral neck fracture in his right hip. A CT scan with intravenous contrast of the abdomen and pelvis revealed marked gallbladder distention (10.4c x 6.4cm) with early intrahepatic biliary dilatation, which was not present in prior studies. No other significant intra-abdominal or intrapelvic pathology was evident (Figure 1).

The patient underwent emergency closed reduction and percutaneous pinning of a right femoral neck fracture, which went well without complications. Further testing was performed after initial laboratory findings and imaging suggested extrahepatic cholestasis (Table 1). Patient's liver function test four months prior to hospitalization revealed mild transaminitis with a normal bilirubin level (Table 2). The results indicated negative for hepatitis, alpha-1 anti-trypsin mutation, anti-nuclear antibodies, and anti-smooth muscle antibodies. Creatine kinase, iron, total iron binding capacity, ferritin, and ceruloplasmin level were all within normal range. He was maintained on intravenous fluids.

**Table 1:** Initial laboratory tests of the patient WBC, white blood cells; RBC, red blood cells; BUN, blood urea nitrogen; AST, aspartate aminotransferase; ALT, alanine transaminase; ALP, alkaline phosphatase

Test	Result	Reference range
WBC	8,330	4,000-10,000
RBC	3.69	4.5-5.5
Hemoglobin	13.6	13.2-16.6 g/dL
Hematocrit	39.7	38.3%-48.6%
Platelet	213	150-317 billion/L
BUN	15	8-23 mg/dL
Creatinine	0.89	0.6-1.2 mg/dL
Calcium, total	9.5	8.5-10.2 mg/dL
Albumin	4.0	3.5-5 g/dL
Globulin	4.0	2.0-3.5 g/dL
AST	206	8-33 U/L
ALT	334	7-56 U/L
ALP	344	40-140 U/L
Bilirubin, total	7.3	0.1-1.2 mg/dL
Bilirubin, direct	6.1	<0.3 mg/dL

**Table 2:** Liver function test results from four months prior to hospitalization to pre-operative day one to post-operative day one (before discharge) AST, aspartate aminotransferase; ALT, alanine transaminase; ALP, alkaline phosphatase

Test	Four months prior to hospitalization	Pre-operative			Post-operative
		Day 1	Day 2	Day 3	Day 1
Albumin	5.2 g/dL	3.6 g/dL	3.1 g/dL	3.2 g/dL	2.9 g/dL
Globulin	5.0 g/dL	3.6 g/dL	2.4 g/dL	2.7 g/dL	2.4 g/dL
AST	59 U/L	132 U/L	73 U/L	122 U/L	110 U/L
ALT	50 U/L	228 U/L	81 U/L	89 U/L	84 U/L
ALP	84 U/L	332 U/L	265 U/L	348 U/L	342 U/L
Bilirubin, total	0.6 mg/dL	8.1 mg/dL	12.7 mg/dL	12.7 mg/dL	10.6 mg/dL

**Figure 2:**
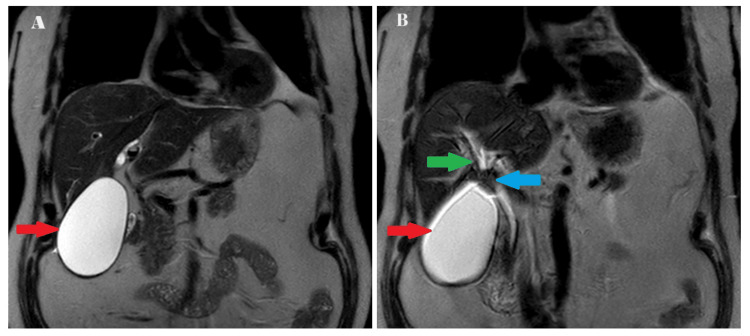
MRCP images of the patient suggesting extrinsic compression of the common bile duct (A) Showing a distended gallbladder pointed with a red arrow. (B) Showing a distended gallbladder pointed with a red arrow, mild central intrahepatic biliary ductal dilatation pointed with a green arrow, non-visualized proximal portion of common bile duct pointed with a blue arrow. MRCP, magnetic resonance cholangiopancreatography

The liver enzyme levels (aspartate aminotransferase (AST), alanine transaminase (ALT), and alkaline phosphatase (ALP)) showed a slight decrease on day one of admission, while bilirubin level remained elevated (Table 2). Magnetic resonance cholangiopancreatography (MRCP) was performed to evaluate the biliary tree, which revealed a distended gallbladder (10.2 x 6.5cm) with probable sludge or stones in the dependent portion, without surrounding inflammation. The distal common bile duct was not dilated, although a portion of the proximal common bile duct was not visualized, raising suspicion of extrinsic compression. Mild central intrahepatic biliary ductal dilatation suggested possible impingement of the proximal common bile duct, potentially secondary to a distended gallbladder, prompting consideration of MS (Figure 2).

The diagnosis of MS was made based on the persistent hyperbilirubinemia, transaminitis, and imaging findings. Subsequently, the patient underwent laparoscopic cholecystectomy, which revealed chronic cholecystitis with cystic duct wall fibrosis most probably due to chronic inflammation, and an intraoperative cholangiogram (IOC) confirmed normal biliary anatomy with no evidence of stones or ductal abnormalities. Histopathology revealed fibrotic tissue with decreased cellularity of the cystic duct with no sign of malignancy of the gallbladder and cystic duct. The patient had no post-surgery complications, and his blood test results showed improvement the next day. Antibiotics were de-escalated. He was discharged a day later with a final diagnosis of hydrops gallbladder and MS type I. Regular follow-up with general surgery and gastroenterology was planned.

## Discussion

MS is a rare condition, which is present in less than 1% of the population [5]. It commonly occurs when a gallstone impacts the flow from the gallbladder, obstructing the common bile duct or hepatic duct and leading to obstructive jaundice. In this case, the obstruction was caused by a distended gallbladder due to fibrosis of the cystic duct, impinging proximal common bile duct leading to type I MS.

In 1982, McSherry et al. categorized MS into type I, caused by gallstones compressing the common hepatic duct, and type II, involving erosion from the cystic duct leading to a cholecystobiliary fistula [6]. Csendes et al. further expanded the classification in 1989 by splitting type II into three subtypes and later introduced type V in 2007 for cholecystoenteric fistulas. Type V was subdivided into Va (gallstone ileus absent) and Vb (gallstone ileus present) [7]. The Csendes classification is currently the most commonly used method. A summary of the classification of MS, from type I to type V, is provided in Table 3 [6,7].

**Table 3:** Classification of MS MS, Mirizzi syndrome

Types		Description
Type I		Compression of the bile duct caused by stones located in Hartmann's pouch or the cystic duct
Type II		A cholecystobiliary fistula involving erosion affecting up to one-third of the bile duct wall's circumference
Type III		A cholecystobiliary fistula involving erosion affecting up to two-third of the bile duct wall's circumference
Type IV		A cholecystobiliary fistula involving erosion affecting the whole of the bile duct wall's circumference
Type V	Va	Development of a cholecystoenteric fistula accompanied without gallstone ileus
	Vb	A development of a cholecystoenteric fistula accompanied by gallstone ileus

MS presents a diagnostic challenge due to its rarity and the nonspecific nature of its symptoms, which can resemble those of other hepatobiliary disorders such as cholangiocarcinoma, primary sclerosing cholangitis, and bile duct strictures [8]. The diagnosis relies on a combination of clinical presentation, laboratory findings, and imaging studies. Patients with MS commonly experience symptoms such as right upper quadrant abdominal pain, elevated liver enzymes, and jaundice, with other symptoms including nausea, vomiting, cholangitis, fever, and anorexia. Asymptomatic cases ranged between 3.7% and 17%, which we observed in the patient [6,9].

Imaging studies play a crucial role in the diagnosis of MS. Ultrasound is often the first imaging modality used and can reveal gallbladder distension, gallstones, and bile duct dilation. However, its sensitivity is limited in detecting the exact nature of the biliary obstruction [5]. CT scans can provide detailed information about the anatomy of the hepatobiliary system and detect complications such as gallbladder perforation or abscess formation [10]. However, MRCP has high sensitivity particularly useful in visualizing the biliary tree and can help identify the site of obstruction and any associated fistulas. It is also useful in diagnosing issues by assessing the extent of pericholecystic inflammation and ruling out other causes like underlying malignancies making it superior to abdominal ultrasonography or CT scans [10].

Endoscopic retrograde cholangiopancreatography (ERCP) is a gold standard invasive procedure, which is used for the diagnosis of MS with a sensitivity of 50%-100% [7]. In addition to diagnosis, it can also aid in managing by assisting with biliary stenting or stone retrieval to relieve obstruction, making it an essential tool for both diagnosing and treating.

The management of MS typically involves surgical intervention. The choice of surgical technique depends on the severity of the obstruction and the presence of a cholecystocholedochal fistula. In type I MS, a simple cholecystectomy may be sufficient. However, in more advanced cases (types II-V), more complex biliary reconstructive procedures may be required [3].

In this case, the patient was diagnosed with MS type 1 by the compression of the bile duct with an enlarged gallbladder as was reported by Williams [9]. He underwent a laparoscopic total cholecystectomy. Despite the chronic cholecystitis and fibrosis observed during surgery, no evidence of malignancy was found in the histopathological examination.

## Conclusions

MS, though rare, should be considered in patients with obstructive jaundice and a history of gallstones. Although MS due to cystic duct fibrosis is less common, in our case, the obstruction was indeed caused by this rare condition, which resulted from a chronic inflammatory process. Imaging studies like MRCP and ERCP are essential for diagnosing this condition and should be considered early. Proper classification aids in determining the right surgical approach, with the intervention being the cornerstone of treatment to relieve biliary obstruction and prevent further complications.
